# Enhanced l-Lysine into 1,5-Diaminopentane Conversion via Statistical Optimization of Whole-Cell Decarboxylation System

**DOI:** 10.3390/polym11081372

**Published:** 2019-08-20

**Authors:** Hanyong Kim, Hah Young Yoo, Nohseong Park, Haeun Kim, Jonghwa Lee, Yesol Baek, Taek Lee, Jong-Min Oh, Jaehoon Cho, Chulhwan Park

**Affiliations:** 1 Department of Chemical Engineering, Kwangwoon University, Seoul 01897, Korea; 2 Department of Biotechnology, Sangmyung University, Seoul 03016, Korea; 3 Department of Electronic Materials Engineering, Kwangwoon University, Seoul 01897, Korea; 4 Department of Chemical and Biomolecular Engineering, Sogang University, Seoul 04107, Korea; 5 Green Materials and Process R&D Group, Korea Institute of Industrial Technology (KITECH), ChonAn 31056, Korea

**Keywords:** decarboxylation, 1,5-diaminopentane, *Hafnia alvei*, l-lysine, statistical optimization

## Abstract

The global lysine companies in the feed industry have steadily built their production facilities due to the high demand for l-lysine in animal farms, and in recent years there have been excessive supply problems and the world market price of l-lysine has fallen. In this study, the conversion of 1,5-diaminopentane (DAP) by decarboxylation of l-lysine was strategically chosen to enhance the value of lysine. The decarboxylation is enzymatically accessible, and *Hafnia alvei*, which is the producer of l-lysine decarboxylase, was applied as a whole-cell form. In the designed whole-cell biocatalytic system, the major four reaction factors were selected by fundamental investigation and then statistical optimization was performed to estimate the optimum condition. The predicted conversion was assessed at about 94.6% at the optimum conditions (125.1 mM l-lysine and 71.5 g/L acetone concentration at 35.2 °C for 8.4 h). Under the determined conditions, DAP conversions by using analytical, feed and industrial crude l-lysine were found to be 98.3%, 92.5% and 72.4%, respectively. These results could be suggested to solve the problem of excessive supplied lysine and also to provide guidance for improved enzymatic conversion by statistical optimization.

## 1. Introduction

**Figure 1:**
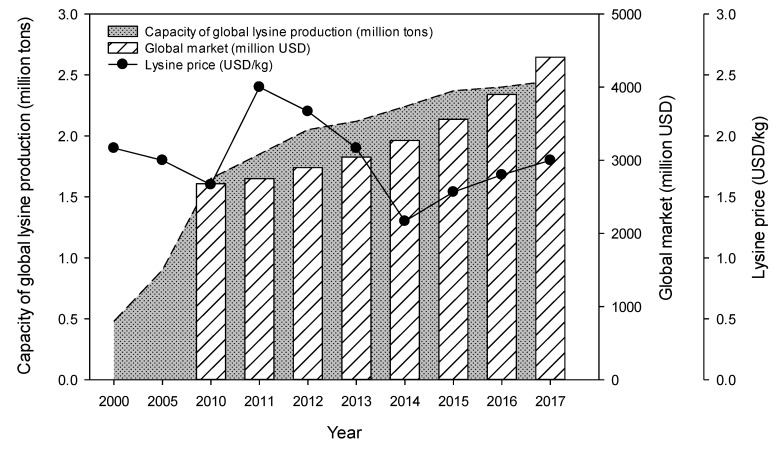
Global production capacity, market and price of lysine.

l-Lysine has been produced steadily nowadays due to the high demand in the animal feed industry. Since l-lysine, an essential amino acid, cannot be synthesized in animals, they should be supplied in food, and the requirements for specific amino acids vary from animal to animal. For instance, modernized pig farms commonly feed relatively large amounts of l-lysine into the diet because of the high requirement of l-lysine in the growth of pigs [1,2]. In particular, the increase in global meat consumption has increased the demand for l-lysine in feed. As a result, many companies have invested in the production of l-lysine and its production has significantly increased in recent years. The l-lysine production in world-wide reached about 2.5 million tons in 2017, and the global market size is increasing by 7% annually (Figure 1). According to the market reports [3,4], the global consumption of l-lysine in 2022 is estimated to be approximately 3.0 million tons and the value of the market is expected to be about $5.6 billion. Since more than half of the lysine market is in Asia and North America, about 70% of the l-lysine demand is supplied by five major producers (Global BioChem, Ajinomoto, ADM, Kyowa Hakko and CJ Biotech Co.) in the region [1,2,3,4,5]. However, the market price of l-lysine is very unstable (about $2.4/kg in 2011, $1.3/kg in 2014 and $1.8/kg in 2017) due to various external factors such as fluctuations in raw material prices, global recession, and oversupply due to excess production. The companies that lose their competitiveness in the lysine industry has become bankrupt or merged due to these problems [5,6]. Therefore, new strategies such as technology development to convert excessively produced lysine into high-value-added materials [7,8], bio-refinery technology using low-cost biomass [9,10] and reduction of manufacturing cost through the process development [11,12] have suggested.

Several studies have been introduced to convert l-lysine as a precursor to a substance such as 1,5-diaminopentane (DAP) or glutaric acid. Applying this process can improve the competitiveness of related industries by suggesting a new strategy of utilizing l-lysine which was overproduced and devaluated [3,4,8]. In particular, DAP has been considered as a potential platform material due to its various applications such as polyurethanes, paints and chelating agents. In addition, DAP can be converted to polyamide 54 and polyamide 510 by biological polymerization, and the properties have similar results compared with petroleum based nylon (polyamide 6 and polyamide 66) [13,14]. Therefore, it is expected that the use of DAP in the polyamide industry will be activated in the near future, and also is expected to be an opportunity for companies with relevant technologies.

Recently, the value of DAP has increased, and the biological technologies which include the production and purification of DAP have been reported [15,16,17]. In general, DAP can be converted by l-lysine decarboxylation and the chemical reaction is described as the removal of the carboxyl group of l-lysine by lysine decarboxylase (EC: 4.1.1.18) and the release of carbon dioxide (CO_2_). Several microorganisms have been reported as producers of l-lysine decarboxylase, and the representative strains are *Corynebacterium glutamicum, Escherichia coli, Hafnia alvei* and *Selenomonas ruminantium* [18,19,20,21,22]. Most of the previous studies have applied genetic manipulation (over-expression) techniques of microbial strains to improve l-lysine decarboxylation. However, the application of engineered strains to industrial fermentation has found a variety of limitations, one of which is an expensive and specific nutritional requirement for microbial culture. Another limitation is that it is difficult to produce high concentrations of DAP since decarboxylation of l-lysine is regulated in vivo and substrates above a certain concentration cannot be accepted into the cells. Thus, because the amount of l-lysine transferred into the cell is limited, the amount of product that can be converted from the substrate is also limited [14,16].

In order to solve the problems caused by microbial fermentation such as low conversion, low substrate uptake and low productivity, our research group has newly designed a whole cell biocatalytic conversion system and have reported it recently. In particular, it found that the use of surfactants and organic solvents in the designed enzymatic system has a significant effect on the conversion of DAP [7]. The aim of this study is to enhance the conversion through the optimization of major factors in the whole cell biocatalytic conversion system. The list and influence of the variables to be optimized were investigated in the fundamental experiment and finally four factors (substrate concentration, reaction temperature, reaction time and acetone concentration) were selected. To determine the maximum DAP conversion, the statistical optimization of four factors was carried out by the response surface methodology (RSM). In addition, analytical l-lysine as well as feed and industrial crude l-lysine were applied to the system as a substrate.

## 2. Materials and Methods 

### 2.1. Materials

Yeast extract peptone dextrose (YPD) broth was purchased from BD Difco (Marvland, MD, USA). l-Lysine, analytical grade (>99.5%) was purchased from Daejung Chemical and Metals Co. Ltd. (Siheung, Korea). l-Lysine, not for analytical grade (feed and industrial crude), was obtained from PKI Co. Ltd. (Gunsan, Korea). 1,5-Diaminopentane and Brij 56 were purchased from Sigma-Aldrich (St. Louis, MO, USA). Acetone was purchased from Junsei (Tokyo, Japan). All chemicals except the lysine (feed and industrial crude) were used above the analytical grade.

### 2.2. Conditions of Whole-Cell Biocatalystic System

The producer of l-lysine decarboxylase, *Hafnia alvei* ATCC9760, was purchased from the American Type Culture Collection (ATCC, Manassas, VA, USA). The culture of *H. alvei* was carried out in a shaking incubator (200 rpm) at 30 °C for 18 h. The cells were harvested by centrifugation at 12,000 × g for 10 min, and then washed with 50 mM phosphate buffer (pH 5.6). The enzyme reaction for DAP conversion was carried out in a 50 mL Erlenmeyer flask (5 mL working volume) at each designed temperature and shaking speed of 200 rpm for each assigned time.

### 2.3. Experimental Design and Statistical Analysis

To maximize the DAP conversion, four major factors of enzyme reaction was statistically optimized by using the response surface methodology (RSM). To build a second order (quadratic) model for the response, 30 experiments were required based on a 5 level 4 factor central composite rotatable design (CCRD), which included 16 factorial, 8 axial, and 6 central points. Table 1 shows the selected factors with 5 coded levels in the central composite design and the experimental factors with ranges were as follows: l-lysine concentration (*X*_1_; 50–250 mM), reaction temperature (*X*_2_; 25–45 °C), reaction time (*X*_3_; 4–12 h) and acetone concentration (*X*_4_; 0–160 g/L). 

Table 2 shows the design of experiments and their response. The DAP conversion was set as a response and the data were analyzed using Design-Expert 7 software (Stat-Ease Inc., USA). The mean values of the RSM were analyzed by a second-order polynomial equation, and the second-order coefficients were generated by regression and stepwise elimination.

The model quality was evaluated by the coefficients of determination (R^2^) and the analysis of variances (ANOVA). The quadratic response surface model was fitted by following Equation (1):

(1) $$Y=\beta_{0}+\overset{4}{\underset{i=1}{\sum }}\beta_{i}X_{i}+\overset{4}{\underset{i=1}{\sum }}\beta_{ii}X_{i}^{2}+\overset{3}{\underset{i=1}{\sum }}\overset{4}{\underset{j=1}{\sum }}\beta_{ij}X_{i}X_{j}$$

where *Y* is the response factor (DAP conversion), *X_i_* and *X_j_* are the *i*^th^ and *j*^th^ independent factor, respectively; *β_0_* is the intercept; *β_i_* is the first order model coefficients; *β_ii_* is the quadratic coefficients for the factor *i*; *β_ij_* is the linear model coefficient for the interaction between factors *i* and *j* [23,24,25].

### 2.4. Conditions of Whole-Cell Biocatalystic System

In order to analyze the DAP concentration, reactants were centrifuged at 9000 × g for 10 min, and then filtration of the supernatant was performed by a hydrophilic syringe filter (Advantec DISMIC-13JP PTFE 0.20 μm, Tokyo, Japan). The components of the filtrate were analyzed using the HPLC system (Agilent 1100, CA, USA) and the assay was carried out by the o-phthaladehyde (OPA) fluorometric method [26]. The HPLC system was equipped with a Zorbax eclipse XDB C18 column, the temperature was maintained at 40 °C, and acetonitrile and 40 mM acetate buffer (pH 5.6) at 40:60 were used as the mobile phase and the flow rate was 1.0 ml/min. The detection wavelength of the 1100 diode array detector (DAD) was 338 nm with a 10 nm bandwidth. The reference wavelength was 390 nm with a 20 nm bandwidth [21]. The conversion of DAP was calculated [7] by the following Equation (2):

(2) $$\mathrm{DAP}\;\mathrm{Conversion}\;\left(\%\right)=\frac{\mathrm{produced}\;\mathrm{DAP}\;\mathrm{concentration}\;\left(\mathrm{mM}\right)}{\mathrm{initial}\;L-\mathrm{lysine}\;\mathrm{concentration}\;\left(\mathrm{mM}\right)}\times 100$$

## 3. Results and Discussion

Theoretically, 1 mole l-lysine can be converted to 1 mole DAP by decarboxylation [22]. *H. alvei*, l-lysine decarboxylase producer, was utilized in the enzymatic conversion system as a whole-cell form. In our previous work, the reaction conditions for the whole-cell biocatalytic conversion system were fundamentally investigated, such as initial l-lysine concentration, reaction temperature and time and additives (detergents and organic solvents) [7]. As a result, it was found that an addition of Brij 56 (non-ionic detergent) significantly affected the enzymatic conversion system. The DAP conversion was achieved over 90% at the initial l-lysine concentration range of 100 to 200 mM, temperature range of 35 to 45 °C, 10% Brij 56 (detergent) and acetone concentration range of 5 to 10% for 8 h reaction.

In order to enhance the DAP conversion, the whole-cell biocatalytic conversion system was statistically optimized based on the previous results. The statistical optimization was performed in accordance with four-factors (*X*_1_, l-lysine concentration; *X*_2_, reaction temperature; *X*_3_, reaction time; and *X*_4_, acetone concentration) specified by the central composite rotatable design. Table 2 lists the experimental factors and the response based on the design of experiments. All 30 of the designed and required experiments were carried out and the coefficients of the model were evaluated by regression analysis for their significance. As a result of ANOVA, non-significant coefficients were selected and excluded on the basis of *P*-value (>0.05), and the most suitable model for statistical use was determined by regression and stepwise elimination. It was represented that three linear coefficients (*X*_1_, *X*_3_, *X*_4_), four quadratic coefficients (*X*_1_^2^, *X*_2_^2^, *X*_3_^2^, *X*_4_^2^) and one cross-product coefficient (*X*_3_*X*_4_) were significant. 

Table 3 shows the ANOVA for the response surface reduced quadratic model. The coefficients of the response surface model were evaluated by Equation (1). One of linear coefficients (*X*_2_) and 5 cross-product coefficients were not significant. However, to minimize error, all the coefficients were considered in the design. A result of ANOVA shows a low lack of fit, and it indicates that the represented model is well within the selected ranges. The final estimated response model equation (based on the coded value) which is excluded the non-significant factors to predict the DAP conversion followed Equation (3):

*Y* = 93.46 − 2.89*X*_1_ + 1.86*X*_3_ − 2.25*X*_4_ − 2.77*X*_1_^2^ − 3.89*X*_2_^2^ − 3.53*X*_3_^2^ − 3.61*X*_4_^2^ − 1.73*X*_3_*X*_4_ (3)

where *Y* is the DAP conversion (%), the response factor. The independent factors, *X*_1_, *X*_2_, *X*_3_, and *X*_4_ mean l-lysine concentration (mM), reaction temperature (°C), reaction time (h) and acetone concentration (g/L), respectively. All *P*-value of coefficients were less than 0.05, and the coefficient of determination (R^2^) was 0.84, thus the model is suitable to sufficiently represent the real relationship among the factors.

**Figure 2:**
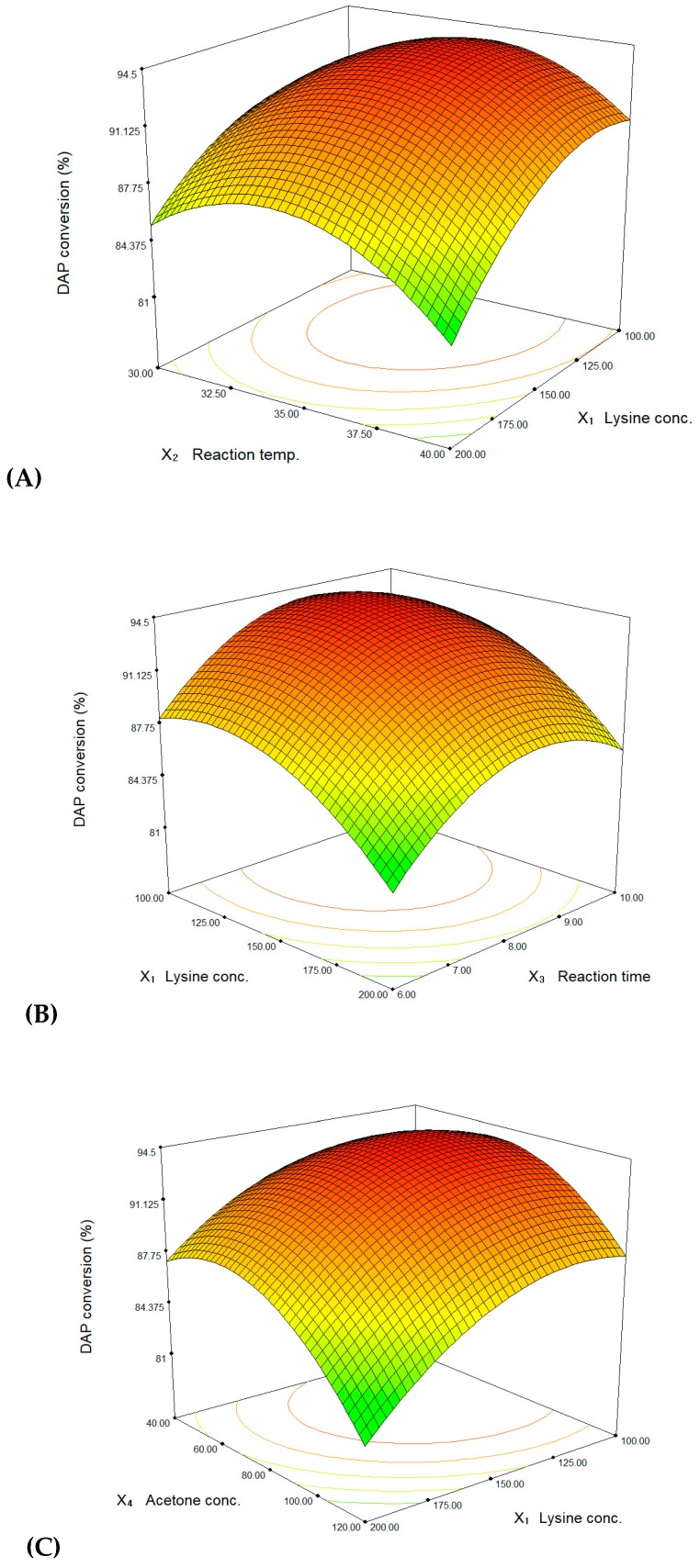

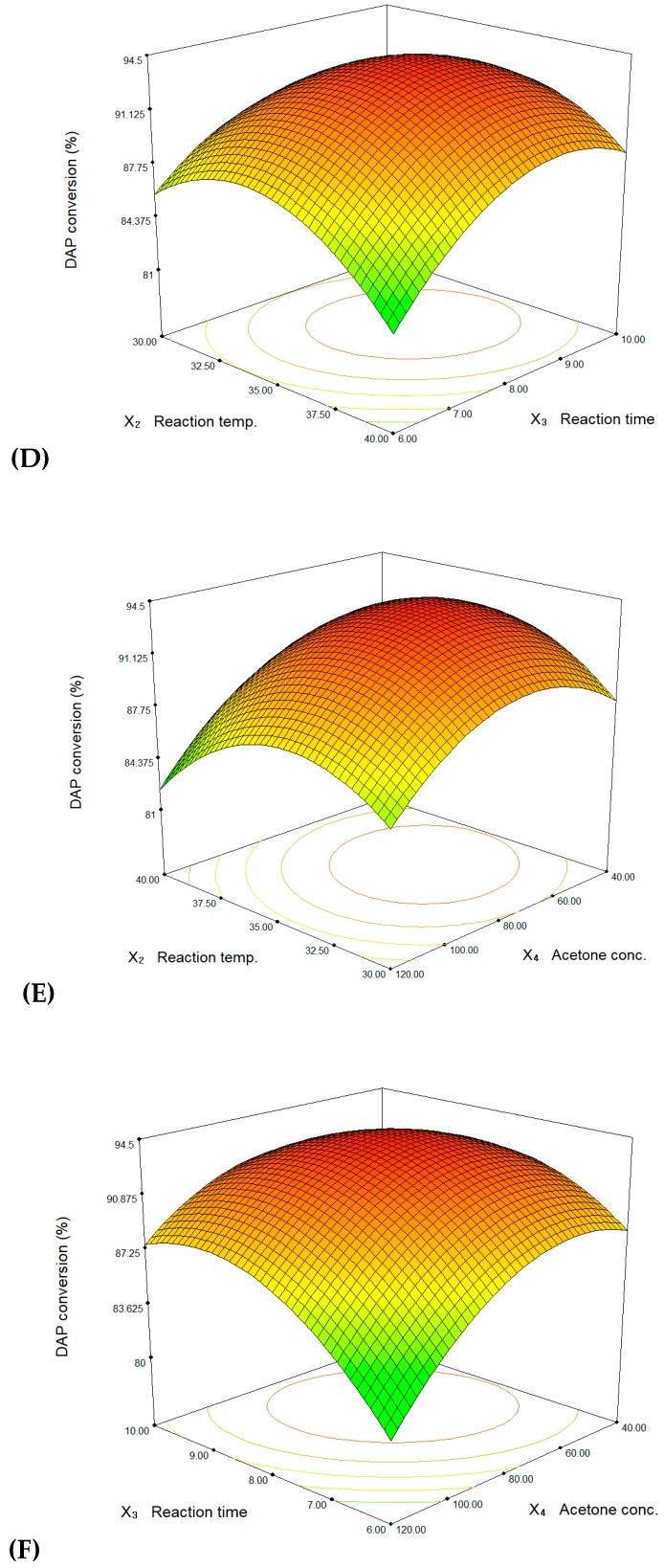
Response surface plots of DAP conversion representing the effect of l-lysine concentration and reaction temperature (**A**), l-lysine concentration and reaction time (**B**), l-lysine concentration and acetone concentration (**C**), reaction temperature and time (**D**), reaction temperature and acetone concentration (**E**), and reaction time and acetone concentration (**F**). Other factors are constant at zero levels, respectively.

The relationship between the reaction factors and the response is represented by the response surface plots (Figure 2) from the predicted model (Equation (3)). Figure 2A shows the effects of l-lysine concentration and reaction temperature on DAP conversion at 80 g/L acetone concentration for 8 h. DAP conversion was maximized (>94%) at the l-lysine concentration range of 100 to 150 mM and temperature range of 32 to 37 °C. Figure 2B shows the effects of l-lysine concentration and reaction time on DAP conversion at 35 °C with 80 g/L acetone concentration. The conversion was maximized (>94%) at l-lysine concentration range of 100 to 150 mM and time range of 7 to 9 h. Figure 2C shows the effects of l-lysine concentration and acetone concentration on DAP conversion at 35 °C for 8 h. The conversion was maximized (>94%) at the l-lysine concentration range of 100 to 150 mM and acetone concentration range of 60 to 80 g/L. Figure 2D represents the effects of reaction temperature and time on DAP conversion at constant levels (150 mM l-lysine and 80 g/L acetone concentration). The high conversion was estimated about 93% at the temperature range of 34 to 36 °C and time of 8 to 9 h. Figure 2E represents the effects of reaction temperature and acetone concentration on the DAP conversion at 150 mM l-lysine concentration for 8 h. The high conversion (>93%) could be achieved by addition of 60 to 80 g/L acetone at the temperature range of 34 to 36 °C. Figure 2F shows the effects of reaction time and acetone concentration on DAP conversion at 35 °C with 150 mM l-lysine concentration for 8 h. The conversion was maximized (>94%) at the acetone concentration range of 60 to 80 g/L and time of 8 to 9 h. The optimum conditions for the whole-cell biocatalytic conversion system were obtained by solving the model equation (Equation (3) using Design-Expert 7 software. In the numerical optimization, the desired goal for DAP conversion was controlled as ‘maximize’ within the designed level (in range) of factors. The estimated optimum conditions by the model equation were 125.1 mM l-lysine concentration (*X*_1_), 35.2 °C temperature (*X*_2_), 8.4 h time (*X*_3_), and 71.5 g/L acetone concentration (*X*_4_). The theoretical DAP conversion was *Y* = 94.6% at the determined conditions. To confirm the prediction by the model, the optimum conditions were applied to DAP production. As a result, the DAP conversion was achieved 98.3 ± 1.2% and it was well within the estimated value of the model equation. Therefore, RSM with a suitable experimental design could be effectively applied to optimize the whole-cell biocatalytic conversion system.

**Figure 3:**
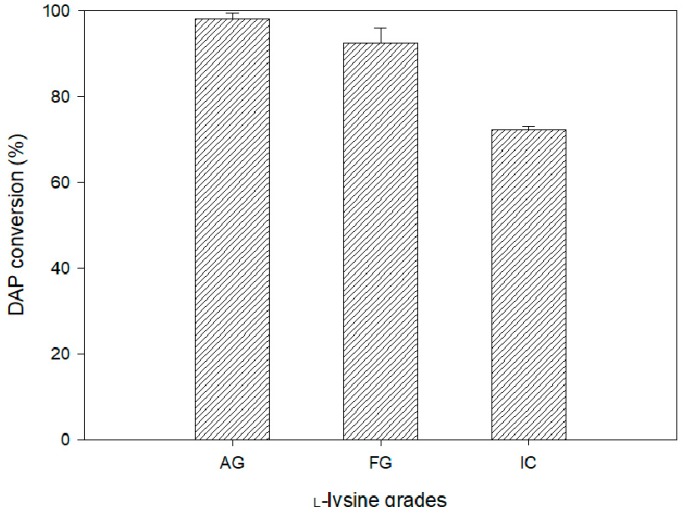
Effect of different grades of l-lysine application on DAP conversion in the determined system with optimum conditions. (AG: >99.5%, analytical grade; FG: ~90%, feed grade; IC: ~50%, industrial crude grade).

In general, production costs are significantly affected by raw materials in the manufacturing process [27,28,29,30,31]. In actual industrial processes, they are trying to use mostly cheap raw materials to lower production costs, however, most of them (low grades or heterogeneous substrate) have a disadvantage of poor purity. As we know, most studies were used analytical grade l-lysine (>99.5% purity) in DAP conversion experiments [15,16,17,18,19,20,21]. However, industrially supplied lysine is produced in the feed grade or bulk chemical, which would require the use of such unrefined lysine for economic reasons in actual DAP industrial processes. Therefore, this study designed a comparative experiment using lysine of a different purity, and the experiments were carried out in the optimized system. In the application of the feed grade l-lysine (FG, about 90% purity), DAP conversion was found to be 92.5 ± 3.6%, whereas, utilization of industrial crude l-lysine (IC, about 50% purity) was achieved at about 72.4 ± 3.7% (Figure 3).

**Figure 4:**
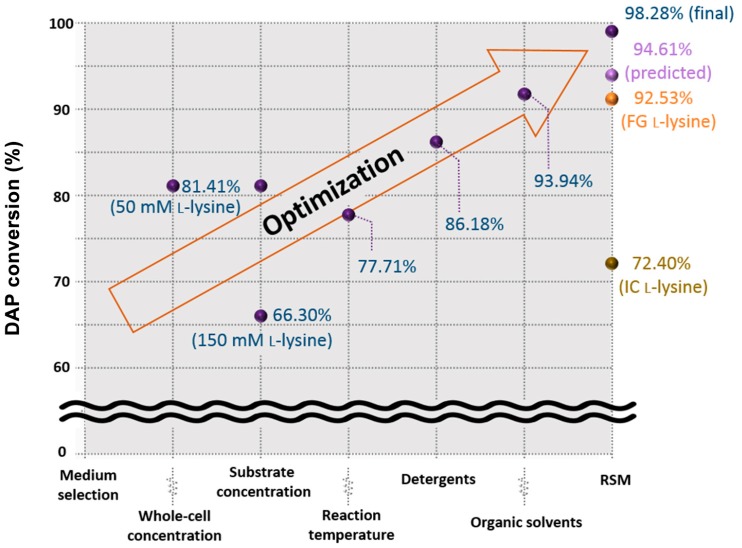
Overall results of DAP conversion by decarboxylation of l-lysine.

Previous and current results of DAP conversion were summarized in Figure 4. Previously, the effect of reaction factors on DAP conversion was investigated step-by-step in order to improve the yield of whole-cell biocatalytic system [7]. First, the effect of enzyme concentration on DAP conversion was investigated and the highest conversion was found to be 81.4% at biocatalyst loading of OD_600_ 4.0. In addition, the DAP conversion according to the initial substrate concentration was 66.3% at the initial l-lysine concentration of 150 mM. The best condition for DAP conversion at a temperature range of 25 to 55 °C was found to be 35 °C (about 77.7% DAP conversion). Next, various surfactants and organic solvents were added to investigate the effect of DAP conversion. The non-ionic detergent, Brij 56, was most effective at 86.2% DAP conversion. The organic solvent showed the highest conversion of 93.9% when acetone was added. Previous studies have used a ‘one factor at time design’ to investigate the effect of reaction conditions. However, the effect of more than two factors on the enzyme reaction system is unknown and requires a lot of experimental trials for research.

Therefore, the current study has effectively derived the optimum conditions of the major reaction factors by RSM, and the predicted DAP conversion was estimated about 94.6%. Interestingly, the actual result shows that the DAP conversion of 98.3% has been achieved and is about 5% improved from the optimal conditions of the previous study. Although the purity is lower, it can be seen that the use of FG and IC, which is advantageous in terms of raw material prices, results in a decrease in DAP conversion. It is presumed that the impurities have adversely affected the enzyme inhibition. As a result, the final conversion from FG and IC achieved about 92.5% and 72.4%, however, the conversions of about 5.8% and 25.9% were lower than those using analytical l-lysine (>99.5% purity), respectively. We have discovered that there are still technical barriers to utilizing IC. In the next study, we will identify the components of impurities in IC and develop strategies for their removal. The finding of this study can be suggested to solve the excessive supply of lysine, and moreover, provide beneficial information on the development of bioconversion processes using whole-cell biocatalysts.

## 4. Conclusions

In this study, to enhance l-lysine into DAP conversion, the reaction conditions for *H. alvei* applied whole-cell biocatalytic system was optimized by using RSM based on 5-level-4-factor CCRD. The most appropriate model of l-lysine decarboxylation was determined by regression and stepwise exclusion of non-significant factors. The optimum conditions were obtained (125.1 mM l-lysine with 71.5 g/L acetone at 35.2°C for 8.4 h) by solving the model equation and the estimated DAP conversion was found to be 94.6%. To confirm the prediction, the determined conditions were applied in the conversion system. As a result, DAP conversion was achieved about 98.3%, indicating that the predictive model is suitable for enzymatic decarboxylation system. In addition, feed (about 90% purity) and industrial crude (about 50% purity) l-lysine were applied as a raw material, and the conversion by using feed and industrial crude l-lysine were achieved about 92.5% and 72.4%, respectively. Therefore, lower grades l-lysine could be applied to the conversion system. This work suggests to solve the excessive supply of l-lysine but also to reduce the process cost via whole-cell biocatalytic conversion system.

## Figures and Tables

**Table 1:** Factors and their values in the central composite design under experimental conditions.

Factors	Symbols	Coded Levels				
		−2	−1	0	1	2
l-Lysine concentration (mM)	*X*_1_	50	100	150	200	250
Reaction temperature (°C)	*X*_2_	25	30	35	40	45
Reaction time (h)	*X*_3_	4	6	8	10	12
Acetone concentration (g/L)	*X*_4_	0	40	80	120	160

**Table 2:** The central composite rotatable design and experimental data for DAP production.

Std.	l-Lysine Conc. (mM), *X*_1_		Reaction Temp. (°C), *X*_2_		Reaction Time (h), *X*_3_		Acetone Conc. (g/L), *X*_4_		DAP Conversion (%)
	Coded	Actual	Coded	Actual	Coded	Actual	Coded	Actual	
1	−1	100	−1	30	−1	6	−1	40	78.99
2	1	200	−1	30	−1	6	−1	40	86.13
3	−1	100	1	40	−1	6	−1	40	84.64
4	1	200	1	40	−1	6	−1	40	79.41
5	−1	100	−1	30	1	10	−1	40	86.63
6	1	200	−1	30	1	10	−1	40	75.39
7	−1	100	1	40	1	10	−1	40	85.05
8	1	200	1	40	1	10	−1	40	80.85
9	−1	100	−1	30	−1	6	1	120	80.00
10	1	200	−1	30	−1	6	1	120	70.53
11	−1	100	1	40	−1	6	1	120	75.65
12	1	200	1	40	−1	6	1	120	63.73
13	−1	100	−1	30	1	10	1	120	78.14
14	1	200	−1	30	1	10	1	120	79.15
15	−1	100	1	40	1	10	1	120	82.12
16	1	200	1	40	1	10	1	120	76.99
17	−2	50	0	35	0	8	0	80	91.32
18	2	250	0	35	0	8	0	80	76.18
19	0	150	−2	25	0	8	0	80	81.19
20	0	150	2	45	0	8	0	80	77.35
21	0	150	0	35	−2	4	0	80	75.87
22	0	150	0	35	2	12	0	80	85.56
23	0	150	0	35	0	8	−2	0	81.24
24	0	150	0	35	0	8	2	160	79.57
25	0	150	0	35	0	8	0	80	92.86
26	0	150	0	35	0	8	0	80	91.39
27	0	150	0	35	0	8	0	80	94.79
28	0	150	0	35	0	8	0	80	95.20
29	0	150	0	35	0	8	0	80	92.45
30	0	150	0	35	0	8	0	80	94.01

**Table 3:** ANOVA for quadratic model and regression statistics.

Source	Sum of Squares	DF	Mean Square	*F*-Value	*P*-Value (Prob > F)
Model	1390.586	8	173.823	13.199	<0.0001
*X*_1_	200.312	1	200.312	15.210	0.0008
*X*_3_	83.038	1	83.038	6.305	0.0203
*X*_4_	122.014	1	122.014	9.265	0.0062
*X*_1_^2^	210.948	1	210.948	16.018	0.0006
*X*_2_^2^	415.768	1	415.768	31.571	<0.0001
*X*_3_^2^	342.168	1	342.168	25.982	<0.0001
*X*_4_^2^	357.374	1	357.374	27.137	<0.0001
*X*_3_ *X*_4_	48.053	1	48.053	3.649	0.0699
Residual	276.558	21	13.169		
Lack of fit	265.600	16	16.600	7.575	0.0173
Pure error	10.957	5	2.191		
Cor. total	1667.144	29			
